# The Spectrum-STI Groups model: syphilis prevalence trends across high-risk and lower-risk populations in Yunnan, China

**DOI:** 10.1038/s41598-020-62208-3

**Published:** 2020-03-25

**Authors:** Eline L. Korenromp, Wanyue Zhang, Xiujie Zhang, Yanling Ma, Manhong Jia, Hongbin Luo, Yan Guo, Xiaobin Zhang, Xiangdong Gong, Fangfang Chen, Jing Li, Takeshi Nishijima, Zhongdan Chen, Melanie M. Taylor, Kendall Hecht, Guy Mahiané, Jane Rowley, Xiang-Sheng Chen

**Affiliations:** 1 Avenir Health, Geneva, Switzerland; 2 Yunnan Center for Disease Control and Prevention, Kunming, China; 3 National Center for STD Control and Prevention, China Centers for Disease Control, Nanjing, China; 4 National Center for AIDS Control and Prevention, Chinese Centers for Disease Control and Prevention, Beijing, China; 5 World Health Organization Regional Office for the Western Pacific, Manila, Philippines; 6 World Health Organization China country office, Beijing, China; 7 World Health Organization, Department of Reproductive Health and Research, Geneva, Switzerland; 8 Centers for Disease Control and Prevention USA, Division of STD Prevention, Atlanta, Georgia USA; 9 Avenir Health, Glastonbury, USA; 10 Independent consultant, London, UK; 11 Chinese Academy of Medical Sciences & Peking Union Medical College Institute of Dermatology, Nanjing, China

**Keywords:** Epidemiology, Population screening

## Abstract

The Spectrum-STI model, structured by sub-groups within a population, was used in a workshop in Yunnan, China, to estimate provincial trends in active syphilis in 15 to 49-year-old adults. Syphilis prevalence data from female sex workers (FSW), men who have sex with men (MSM), and lower-risk women and men in Yunnan were identified through literature searches and local experts. Sources included antenatal care clinic screening, blood donor screening, HIV/STI bio-behavioural surveys, sentinel surveillance, and epidemiology studies. The 2017 provincial syphilis prevalence estimates were 0.26% (95% confidence interval 0.17–0.34%) in women and 0.28% (0.20–0.36%) in men. Estimated prevalence was 6.8-fold higher in FSW (1.69% (0.68–3.97%) than in lower-risk women (0.25% (0.18–0.35%)), and 22.7-fold higher in MSM (5.35% (2.74–12.47%) than in lower-risk men (0.24% (0.17–0.31%). For all populations, the 2017 estimates were below the 2005 estimates, but differences were not significant. In 2017 FSW and MSM together accounted for 9.3% of prevalent cases. These estimates suggest Yunnan’s STI programs have kept the overall prevalence of syphilis low, but prevalence remains high in FSW and MSM. Strengthening efforts targeting FSW and MSM, and identification of other risk populations e.g. among heterosexual men, are critical to reduce syphilis.

## Introduction

Spectrum-STI is a statistical trend-fitting model designed for countries to estimate trends over time in the prevalence and incidence of three sexually transmitted infections (STIs): syphilis (etiologic agent: *Treponema pallidum* subspecies *pallidum*); gonorrhea (*Neisseria gonorrhoeae*); and chlamydia (*Chlamydia trachomatis*) from available prevalence data after standardizing for diagnostic test, location and age and weighted to reflect representativeness. The model builds on the Spectrum suite of health policy planning and surveillance models developed by Avenir Health^1^. The latest version of Spectrum-STI, the Spectrum-STI Groups model, allows for estimates to be generated for different higher-risk and lower-risk groups in a population and for these to be combined to form an overall population estimate. In November 2018, Spectrum-STI Groups was piloted in a 3-day workshop held in Kunming, Yunnan’s capital, organized by the Yunnan provincial Center for Disease Control and Prevention (CDC) and the World Health Organization (WHO).

Yunnan province is located in the south-west of China and in 2017 had a population of just under 46 million (3.4% of China’s population). It is one of three provinces chosen by China’s National Health and Family Commission and UNICEF to pilot validating elimination of mother-to-child transmission of HIV, syphilis and Hepatitis B. STI control in Yunnan is focused primarily on Female Sex Workers (FSW), Men who have Sex with Men (MSM), Intravenous Drug Users (IDU), and STI clinic attendees and includes health education, condom promotion, free testing for HIV, syphilis and gonorrhea, and free treatment. The STI control budget doubled between 2000 and 2017^2^ and syphilis prevention and treatment efforts have been expanded. Screening has been scaled-up using HIV testing facilities, methadone substitution and detoxification centers, antenatal care clinics (ANC) and hospitals. Syphilis partner notification and referral, and standardized treatment, have been incentivized by performance-based compensation to clinics. This has resulted in a five-fold increase in the number of FSW, MSM, individuals using HIV voluntary counselling and testing facilities and methadone maintenance clients tested for syphilis between 2010 and 2017, reaching 7.83 million people including 20,400 higher-risk individuals screened in 2017 alone. By 2015 testing rates were estimated to be 80% in FSW and 95% in VCT and ANC clients^2^. Among those diagnosed with syphilis, the rate of standard treatment has been above 80% since 2014^2^.

STI surveillance in Yunnan includes etiological case reporting by hospitals and all other institutions that provide STI diagnosis to China’s infectious disease prevention and control information system. These data are collated and reported electronically, on a continuous basis. In addition, in collaboration with the HIV department, there is a sentinel surveillance system measuring syphilis serology annually in FSW and MSM. The surveillance in FSW focuses on women who self-identify as FSW and around 6,000 FSW have been sampled annually since 2005 from 16 sites across the 16 prefectures in Yunnan. Surveillance in MSM focuses on self-identified MSM and around 3,000 men have been sampled annually since 2010 from 14 sites (Table 1). These surveillance data are complemented by ad hoc, typically smaller-scale research studies done in specific population groups.

**Table 1:** Syphilis prevalence data from Yunnan province.

Population group	Population type	Year, midpoint	Diagnostic test	N positive	N tested	Prevalence (%)	Weight (%)	Location/Sites	Source
Lower-risk women	ANC: Survey	2005	RPR & TPHA	4	3,242	0.12	25	4 national surveillance sites within Yunnan province (Kunming, Qilin, Longyang and Gejiu)	^16^
		2006		15	3,241	0.46	25		
		2007		3	2,702	0.11	25		
		2008		12	3,215	0.37	25		
		2009		0	3,041	0.00	25		
		2010		4	1,599	0.25	25		
		2011		5	1,600	0.31	25		
		2012		5	1,600	0.31	25		
		2013		1	1,600	0.06	25		
		2014		9	1,600	0.56	25		
		2015		4	1,600	0.25	25		
		2016		6	1,600	0.38	25		
		2017		6	1,596	0.38	25		
	ANC: Routine screening	2011	RPR & TPHA	239	637,241	0.04	0	All 16 prefectures and 129 counties	^2^
		2012		690	786,334	0.09	0		
		2013		1,032	753,696	0.14	0		
		2014		1,338	761,945	0.18	0		
		2015		1,360	730,474	0.19	99.66		
		2016		1,879	888,171	0.21	99.92		(Unpublished) program data, Yunnan CDC, Kunming, Yunnan
		2017		1,974	814,401	0.24	99.95		
	Blood Donor Screening	2008	TPHA alone	120	29,584	0.41	43.75	7 prefectures (Chuxiong, Nujiang, Wenshan, Xishuangbanna, Yuxi, Diqing and Qujing)	^17,18^
		2009		132	32,465	0.41	43.75		
		2010		139	34,310	0.41	43.75		
		2011		141	35,771	0.39	43.75		
		2012		165	39,956	0.41	43.75		
		2013		159	38,165	0.42	43.75		
		2014		142	42,835	0.33	43.75		
		2015		179	45,849	0.39	43.75		
		2016		241	52,006	0.46	43.75		
		2017		238	55,471	0.43	43.75		
	Premarital Screening	2001	TPHA alone	24	3,742	0.34	6.25	1 site (urban and rural)	^19^
Lower-risk men	Blood Donor Screening	2008	TPHA alone	217	43,020	0.50	43.75	7 prefectures (Chuxiong, Nujiang, Wenshan, Xishuangbanna, Yuxi, Diqing and Qujing)	^17,18^
		2009		244	47,547	0.51	43.75		
		2010		220	52,385	0.42	43.75		
		2011		229	56,517	0.41	43.75		
		2012		297	62,171	0.48	43.75		
		2013		236	56,320	0.42	43.75		
		2014		252	61,675	0.41	43.75		
		2015		275	65,825	0.42	43.75		
		2016		339	73,984	0.46	43.75		
		2017		317	76,685	0.41	43.75		
	Premarital Screening	2001	TPHA alone	24	3,742	0.34	6.25	1 site (urban and rural)	^19^
Lower-risk Women + Men pooled	Blood Donor Screening	2008	TPHA alone	39	9,102	0.43	6.25/0*	1 prefecture (Dehong)	^20^
		2009		29	10,034	0.29	6.25/0*		
		2010		36	11,622	0.31	6.25/0*		
		2011		35	13,302	0.26	6.25/0*		
		2012		53	13,164	0.40	6.25/0*		
		2013		39	12,295	0.32	6.25/0*		
FSW	FSW: Study	2006	RPR & TPHA	55	737	7.46	10	1 site (Kaiyuan City)	^21^
	FSW: Study	2006	RPR & TPHA	12	96	12.50	20	2 sites (Kafang & Laochang towns)	^50^
	FSW: Study	2007	RPR & TPHA	27	397	6.80	10	1 site (Kaiyuan City)	^26^
	FSW: Study	2007	RPR & TPHA	32	270	11.85	10	Detaining education centre, urban	^28^
FSW	FSW: Study	2010.5	RPR & TPHA	3	201	1.49	10	1 site (Kaiyuan City)	^27^
	FSW: Study	2011	RPR & TPHA	113	1,775	6.37	10	1 site (Kaiyuan City)	^53^
	FSW: Study	2012	RPR & TPHA	62	734	8.45	10	1 site (Kaiyuan City)	^23^
	FSW: Study	2012.5	RPR & TPHA	44	225	19.56	40	4 sites (Dali, Jinghong, Kaiyuan and Menghai cities)	^24^
	FSW: Study	2017	RPR & TPHA	13	423	3.07	10	1 site (Kunming City)	^29^
	FSW: Sentinel	2005	RPR & TPHA	107	5,849	1.83	100	All 16 prefectures	^16^
		2006		50	5,396	0.93	100		
		2007		92	5,782	1.59	100		
		2008		77	5,909	1.30	100		
		2009		71	6,225	1.14	100		
		2010		135	5,960	2.27	100		
		2011		113	6,026	1.88	100		
		2012		146	6,047	2.41	100		
		2013		88	6,464	1.36	100		
		2014		68	5,495	1.24	100		
		2015		54	5,836	0.93	100		
		2016		104	6,336	1.64	100		
MSM	MSM: Sentinel	2010	RPR & TPHA	44	1,169	3.76	87.5	14 prefectures	^16^
		2011		21	1,556	1.35	87.5		
		2012		65	2,613	2.49	87.5		
		2013		83	2,992	2.77	87.5		
		2014		91	3,206	2.84	87.5		
		2015		103	2,983	3.45	87.5		
		2016		106	2,863	3.70	87.5		
	MSM: Study	2009	TPHA alone	62	825	7.52	10	1 site (Kunming City)	^30^
	MSM: Study	2010	RPR & TPHA	6	305	1.97	10	1 site (Dali prefecture)	^33^
	MSM: Study	2014	RPR & TPHA	34	300	11.33	10	1 site (Kunming City)	^32^
	MSM: Study	2008	RPR & TPHA	37	266	13.91	10	1 site (Kunming City)	^31^
		2009		40	307	13.03	10		
		2010		36	258	13.95	10		

Pre-2015 ANC routine screening data were not included in the default estimation owing to a change in the ANC screening algorithm in 2015 from Treponema pallidum particle agglutination assay (TPPA) and RPR with an RPR threshold of 1:8, to a positive result from a Treponema-based rapid point-of-care test followed by RPR confirmation with no threshold, and an expansion in the number of sites tested from 2011 to 2015.

A number of the studies in FSW venues were cohort studies with repeated measurements and where later rounds probably resampled the same individuals after they had been treated. For these studies only the 1^st^ round of data were included in the Spectrum estimations.

*6.25% is the default weight for men. These data were not included in default the estimate for women, owing to the large number of other women-specific data points.

Abbreviations: RPR = Rapid Plasma Reagin test; TPHA = Treponema pallidum hemagglutination assay; RPR&TPHA = dual positivity on both RPR and TPHA.

This paper presents the syphilis prevalence trend estimates for 2005 to 2017 generated using Spectrum-STI, for four population groups in Yunnan: Lower-risk women, Lower-risk men, FSW and MSM. These four estimates were then combined into provincial syphilis estimates for all 15–49 year old women and men. Findings are discussed with a view to how Spectrum-STI can be used by national and provincial HIV/STI programs to inform surveillance, evaluation and planning.

## Methods

### Prevalence data

Syphilis prevalence data were identified through a PubMed search of studies published in 2000 or later with the key words ‘China’ AND (‘sexually transmitted infection’ OR ‘sexually transmitted disease’ OR ‘syphilis’), a search of the Chinese scientific literature using these and related key words (using the search systems: www.cnki.net, www.cqvip.com and wanfangdata.com.cn), and back-tracking references of relevant English and Chinese language articles. These data were supplemented by published and unpublished/program data identified by STI experts during and in the 4 months after the November 2018 workshop (Table 1). Only studies where the majority of samples were from Yunnan Province and collected in 2000 or later were included in the analysis.

Data were collected for the following populations:

- Lower-risk women: ANC routine screening (program data), ANC sentinel surveillance surveys, blood donor screening, community surveys, obstetrics & gynaecology patients and pre-marriage screening;
- Lower-risk men: Blood donor screening, pre-marriage screening;
- FSW: Annual sentinel surveillance surveys, ad hoc research studies. Data from FSW seeking care in STD clinics and re-education centers were not included;
- MSM: Annual sentinel surveillance surveys and ad hoc studies.

### Spectrum-STI model

Spectrum-STI is a statistical model that fits time trends in national or provincial adult STI prevalence and incidence, based on STI prevalence data^3,4^. In the fall of 2018, the model was expanded to incorporate different population groups (Spectrum-STI versions 5.73 and onwards) and is available online **(**https://avenirhealth.org/software-spectrum.php). The updated model was piloted in Yunnan province at a workshop in November 2018.

Provincial estimates were generated for four populations – Lower-risk women, lower-risk men, FSW and MSM. The estimates for the four populations were combined, based on their estimated contribution to the provincial population, to form provincial estimates. The adult (15–49 years) female and male population sizes for Yunnan province were created by the Division of Epidemiology, National Center for AIDS/STD Control and Prevention, Chinese Center for Disease Control and Prevention and Avenir Health, based on China’s 2000 and 2010 national population censuses^5^ and Statistical Yearbooks^6^ with their year-on-year projections extrapolated through 2017.

FSW were assumed to account for 0.30% of women 15–49 years of age and MSM for 0.80% of men 15–49, and these percentages were assumed to remain constant over time. The FSW size estimate was based on service statistics of outreach to FSW over 2016–2017 reported to China’s disease prevention and control information system by all 129 counties in Yunnan and is in line with the UNAIDS estimate for Asia of FSW representing an average of 0.35% of adult women^7–9^. The MSM size estimate was based on a community household survey conducted in 2 prefectures of Yunnan in 2014^10^ and is similar to results from two cluster-sampled surveys in male students from all 16 prefectures in Yunnan in 2005 and 2015 in which 0.7% and 0.9% self-reported MSM behavior^11^. Lower-risk women and men population sizes estimates were then derived by subtracting the estimated FSW and MSM populations from the total population (Table 2).

**Table 2:** Population sizes, estimated syphilis prevalence (default estimates) and share of provincial prevalent cases in 4 adult (15–49 years) sub-populations in Yunnan province, 2005 and 2017.

Population group	Population size		Prevalence % (95% Confidence Interval)		Share of all prevalent cases
	2005	2017	2005	2017	2017
Lower-risk women	12,077,922	12,148,877	0.26 (0.11–0.47)	0.25 (0.18–0.35)	45%
Lower-risk men	12,893,877	13,135,136	0.26 (0.18–0.37)	0.24 (0.17–0.31)	46%
FSW	36,343	36,556	1.93 (1.34–2.77)	1.69 (0.68–3.97)	0.91%
MSM	103,983	105,929	6.84 (1.78–13.82)	5.35 (2.74–12.47)	8.4%
**All women**	**12,114,265**	**12,185,433**	**0.26 (0.18–0.35)**	**0.26 (0.17–0.34)**	**46%**
**All men**	**12,997,860**	**13,241,065**	**0.32 (0.24–0.40)**	**0.28 (0.20–0.36)**	**54%**
**All women and men**	**25,112,125**	**25,544,343**	**0.29 (0.22–0.36)**	**0.29 (0.22–0.36)**	**100%**

### Standardizing and weighting prevalence data

Prevalence data from each study were standardized before trend fitting to ensure that they reflected active syphilis, the stage relevant for disease transmission, defined as concurrent positivity on both a non-treponemal (e.g., Rapid Plasma Reagin (RPR) or Venereal Disease Research Laboratory (VDRL) test) and a treponemal test. This adjustment used the same approach and parameters as the WHO 2016 STI estimates^4,12,13^. For lower-risk men and women each study was assigned a weight reflecting how representative it was of the particular population group (Table 1). For FSW and MSM, prevalence trends were estimated separately for the provincial sentinel surveillance system data and for the ad-hoc studies. The two trend estimates were then combined, with the provincial surveillance trend assigned a weight of 94% (reflecting an average 15 out of the province’s 16 prefectures sampled) to account for the greater reliability of these data and the ad hoc studies trend a weight of 6% (reflecting a typical 1 of 16 prefectures sampled).

### Syphilis trend estimates

Prevalence trends were estimated for each population group by fitting the standardized and weighted prevalence data using segmented polynomial regression. Segmented polynomials were fitted to the incidence underlying prevalence, assuming an average duration of infection for syphilis in both women and men of 4.1 years^13^. The fitted incidence curve was a second-order spline with a maximum of 2 knots, selected using the Akaike Information Criterion^4^.

### Uncertainty intervals

Uncertainty bounds for each population group were calculated by bootstrapping^3^. When combining the results to generate the provincial estimates we assumed there was no uncertainty in population sizes. Differences between years were considered statistically significant if the respective 95% confidence intervals did not overlap.

### Sensitivity analysis

Univariate sensitivity analyses looked at the impact of restricting the eligible data by altering the weights attached to individual data points and to data types available within each group. We also looked at the effect of changing the relative population sizes of FSW and MSM, using UNAIDS regional estimates for Asia or all low- and middle-income countries^9,14^ instead of Yunnan’s population size estimates. More general assumptions underlying the Spectrum-STI model have been addressed in sensitivity analyses in earlier publications^3,4,15^.

## Results

### Prevalence data

Table 1 presents the identified data that met the study entry criteria, and the default weights assigned to each data point. A total of 82 syphilis data points from Yunnan province were identified between 2000 and 2017 from ANC women, blood donors, pre-marital screening, FSW and MSM.

#### Lower-risk women

31 data points were identified from ANC routine screening (2011 to 2017), ANC sentinel screening in four cities that were part of China’s national STI surveillance (2005 to 2017)^16^, blood donor screening (7 prefectures from 2008 to 2017, a period when donor eligibility criteria were stable)^17,18^, and pre-marriage compulsory syphilis screening (1 prefecture in 2001, covering urban and rural areas within 1 prefecture^19^).

#### Lower-risk men

11 data points were identified from blood donor screening (7 prefectures from 2008 to 2017, a period when donor eligibility criteria were stable)^17,18^, and pre-marriage compulsory syphilis screening (1 prefecture in 2001, covering urban and rural areas^19^).

#### Lower-risk men and women

6 data points were identified from blood donors where no breakdown by sex was provided (1 prefecture from 2008 to 2013)^20^.

#### FSW

21 data points were identified from the annual sentinel surveillance surveys (16 prefectures from 2005 to 2016^16^) and studies (9 studies conducted between 2006 and 2017^21–29^).

#### MSM

13 data points were identified from the annual sentinel surveillance surveys (14 prefectures from 2010 to 2016^16^) and studies (6 studies conducted between 2009 and 2014^30–33^).

For both FSW and MSM, prevalence in the research studies were more diverse and, on average, higher than in the sentinel surveillance. This may reflect a bias in the sentinel surveillance data towards lower prevalence if FSW and MSM tested and treated in previous rounds are re-sampled. Or it may be that the research studies over-sampled populations in ‘hot-spot’ areas where prevalence is unrepresentatively high.

### Spectrum-STI estimates

**Figure 1:**
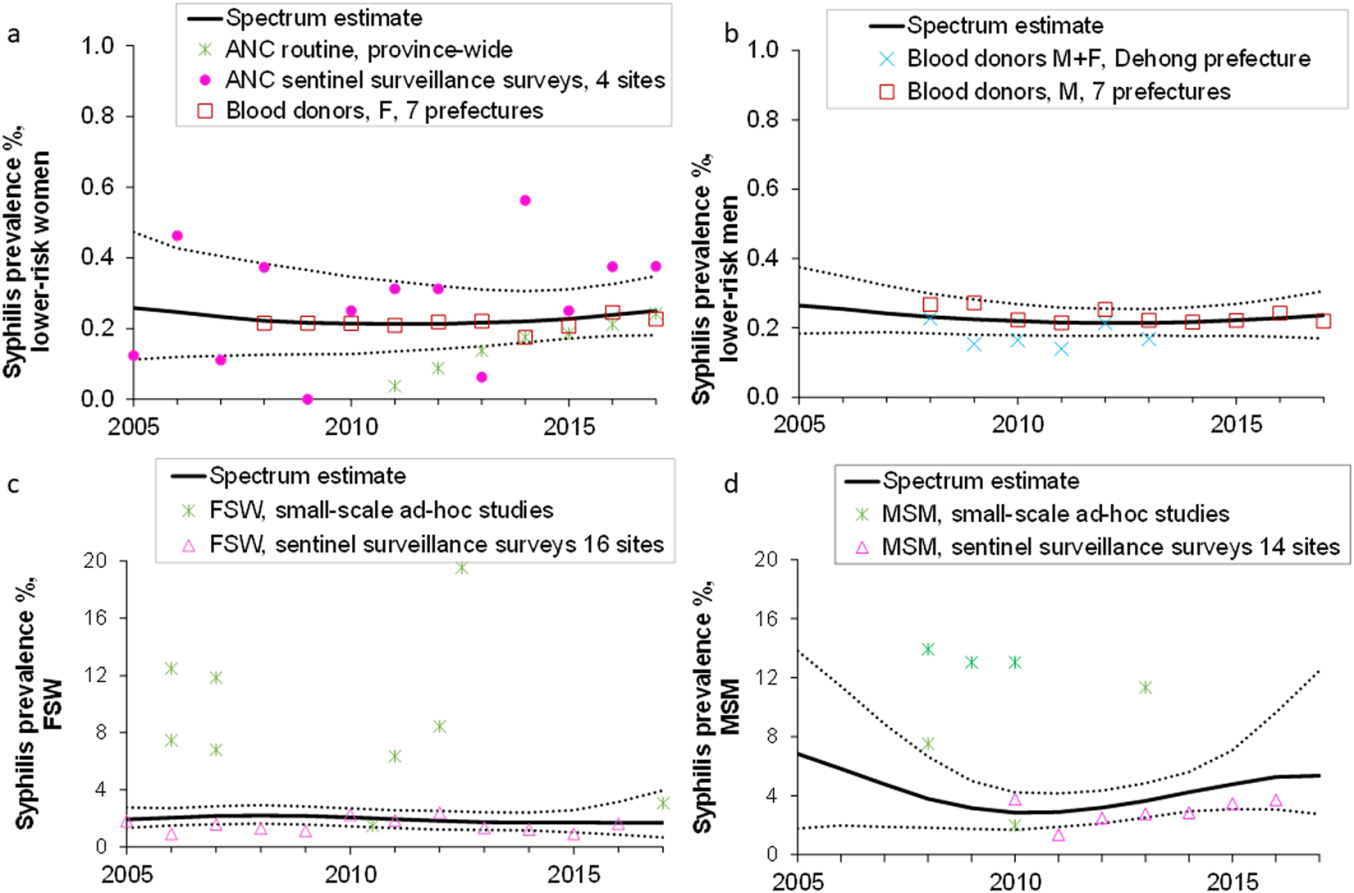
Prevalence data and estimates for active syphilis, Yunnan province, 2005–2017: (**a**) Lower-risk women; (**b**) Lower-risk men: (**c**) Female Sex Workers; (**d**) Men who have sex with men. Prevalence estimates shown are after adjustment for diagnostic test performance. Solid lines are the best estimates; dotted lines the corresponding 95% confidence interval. The blood donor screening data that could not be disaggregated by sex; Table 1) were used in the estimation for lower-risk men, as this group had few other data available (panel b) and the majority of donors in this dataset (56%) were men. In contrast, they were not used in the estimation for lower-risk women, for whom there were many more data points (panel a).

Figure 1 shows the Spectrum-STI estimations for 2005 to 2017 based on the default weightings for the four population groups. The Spectrum-STI trend estimates were restricted to 2005 to 2017, as only one study was identified between 2000 and 2005^19^. In 2017 the estimated prevalence in lower-risk women was 0.25% (0.18–0.35%) and in lower- risk men 0.24% (0.17–0.31%). The prevalence in FSW in 2017, 1.69% (0.68–3.97%) was 6.8-fold greater than in lower- risk women and the prevalence in MSM 5.35% (2.74–12.47%) was 22.7-fold greater than in lower- risk men (see Table 2). Comparing the prevalences in 2005 and 2017, the 2017 estimates were lower than the 2005 estimates for all four population groups, but the 95% confidence intervals overlapped suggesting the differences were not significant.

The estimated prevalence for all women between the ages of 15 and 49 in Yunnan province is almost identical to the estimate for lower-risk women (Table 2) reflecting the small proportion of FSW in Yunnan’s overall women’s population (0.30%). FSW and MSM together accounted for 0.9% and 8.4% of prevalent cases, respectively, totaling 9.3% of all prevalent cases (Table 2).

In 2017, 46% of prevalent cases were in women and 54% in men. The larger share of men primarily reflects the relative contribution of FSW and MSM. FSW accounted for 0.9% of prevalent cases (Table 2), whilst MSM, with their larger share of the male population and higher prevalence, accounted for 8.4%.

### Sensitivity analysis

**Figure 2:**
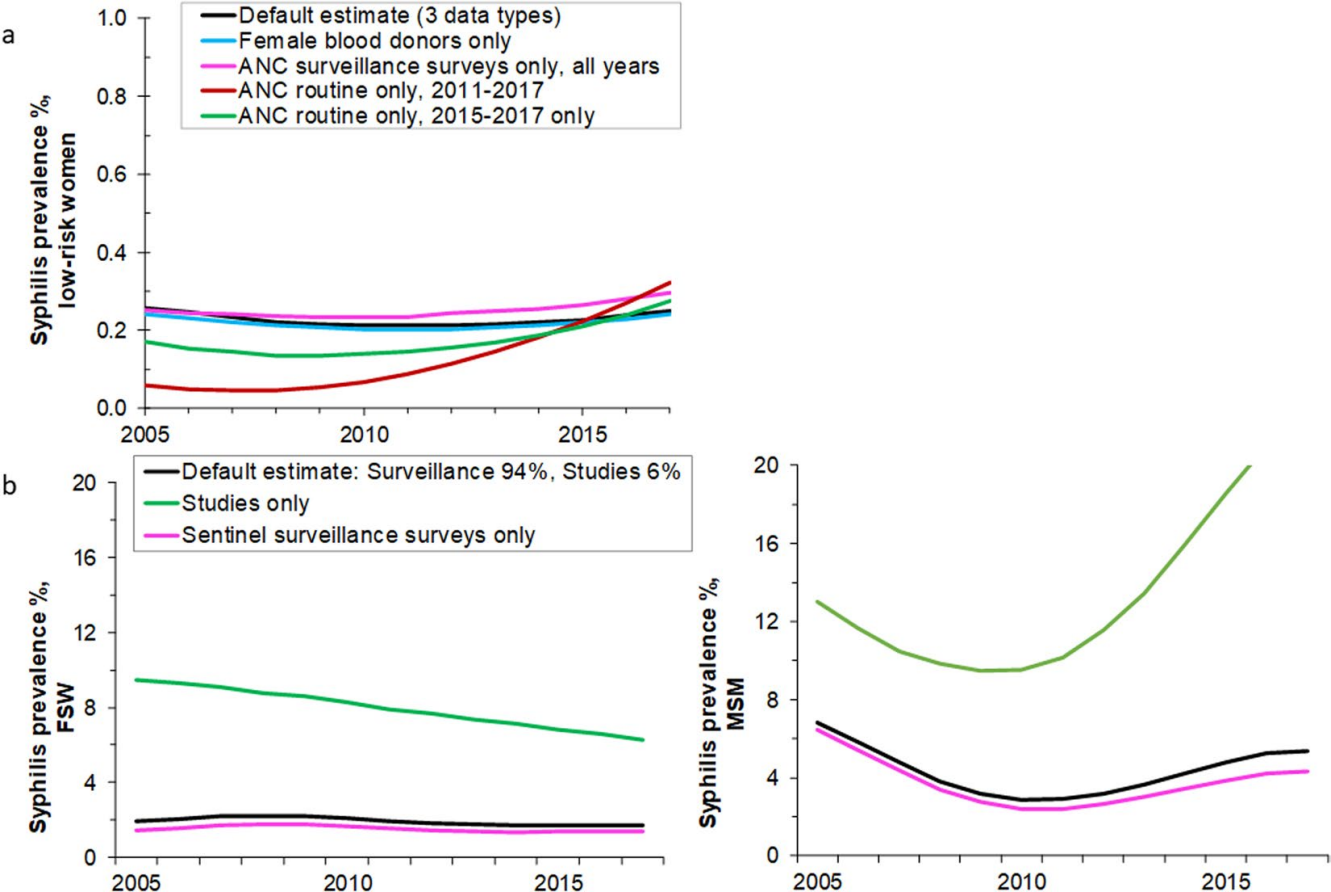
Syphilis prevalence estimates under alternative data inclusion criteria, for (**a**) Lower-risk women, (**b**) FSW and (**c**) MSM. Series titles shown in panel b also apply to panel c.

The effect of altering the prevalence data included in the trend analysis are illustrated in Fig. 2. Figure 2a is for lower-risk women and compares the default estimate that combined three data types, with an estimate based only on (i) routine ANC data, (ii) ANC sentinel surveillance data, or (iii) female blood donor data. The trend estimates for ANC surveillance data and for blood donor data are both similar to the default estimate, with a fairly stable prevalence (Fig. 2a). The ANC routine data only, however, suggest prevalence has increased but this is based on only 3 years of data (2015 to 2017) as the pre-2015 routine ANC measurements used a stricter case definition (threshold titer for RPR test) and covered fewer sites. If the 2011–2014 routine ANC data are added to the analysis (Fig. 1a), the increase is more marked (Fig. 2a).

For FSW (Fig. 2b) and MSM (Fig. 2c) the trend estimates based on the ad hoc studies were much higher than those based on sentinel surveillance surveys alone. For FSW, the estimates based on the sentinel surveillance surveys suggest that between 2005 and 2017 prevalence was stable, whilst those based on the ad hoc studies suggest a steady decline.

For MSM, the estimates based on the sentinel surveillance surveys suggest that prevalence fell between 2005 and 2010 but has been stable or increasing slightly since then. The trend estimate based on the ad-hoc studies, in contrast, shows a strong decline in prevalence followed by a partial more recent increase.

In the default estimates presented above, we assumed 0.30% of the female population were FSW, which is in line with the UNAIDS’ median estimate for the Asian region of 0.35%^8,9^. If the FSW group size is increased to the UNAIDS median across all low- and middle-income countries^8,9^ of 0.67%, the share of FSW in overall adult syphilis prevalence in 2017 increased from 0.91% to 2.0%, and overall female prevalence from 0.256% to 0.263%. If the group size is increased to UNAIDS’ upper limit estimate for the Asian region of 2.33%^8,9^, then FSW account for 6.2% of the overall adult syphilis prevalence in Yunnan, and overall female prevalence would increase to 0.28%.

Finally, increasing the proportion of MSM from the Yunnan estimate of 0.80% to the UNAIDS’ median regional estimate for Asia^9,14^ of 1.69% of the overall male population, the share of MSM in overall adult syphilis prevalence increased from 8.4% to 16%, and the male prevalence estimate from 0.28% to 0.31%.

## Discussion

This study illustrates the functionality and relevance of the Spectrum-STI Groups model for estimating trends in syphilis prevalence across distinct population groups. The model was piloted at a workshop in Yunnan Province that brought together individuals from different implementing and supporting organizations to discuss the strengths and limits of the available local prevalence data and to identify additional data sources. The workshop looked at three infections but, owing to a lack of historical data for gonorrhea and chlamydia for which a prevalence study was just ongoing, we decided to focus this analysis on syphilis.

The 2017 syphilis prevalence estimate in Yunnan for women 15 to 49 was 0.26% (0.17–0.34%) and for men 0.28% (0.20–0.36%). These levels are low compared to other East and Central Asia countries^15^, and relative to countries with similar per capita incomes^34–36^. Given the low prevalence, Yunnan is in a position where it could meet the WHO criteria for eliminating congenital syphilis, provided coverages of ANC attendance, of ANC-based syphilis screening and of syphilis treatment among women diagnosed during ANC are all 95% or higher^37^.

The estimates for all four population groups were lower in 2017 than in 2005, although the differences were not significant. This suggests that the province’s targeted HIV/STI response, which includes free syphilis (and HIV) screening, alongside free counselling and prevention services has helped contain the spread of syphilis during a time of social and economic change and liberalization. For FSW, the estimated decline is consistent with the national FSW surveillance data from sites in other provinces of China followed since 2005^38^. For MSM the results are consistent with the overall decline in syphilis prevalence observed among male patients of STD clinics in Yunnan^2^. The decline is also consistent with the drop in test positivity rates in routine provincial syphilis case reporting, from 0.20% in 2012 to 0.14% in 2017, while numbers tested annually increased 3-fold^2^. Still, based on the estimated trends, Yunnan province is not on track to reach the strong decline required to meet the global target in the WHO Global Health Sector Strategy for STI control, of a 90% reduction in the incidence of syphilis between 2018 and 2030^39^.

For FSW and MSM there were marked differences between the estimates based on the provincial sentinel surveillance data and those based on the small-scale ad hoc studies. The former was, on average, much lower, which may reflect the fact that individuals who are part of the sentinel surveillance system are more likely to be screened and treated and as a result have, on average, a shorter duration of infection.

FSW and MSM together accounted for 9.3% of Yunnan’s estimated prevalent cases in 2017. This is lower than the share of these key groups in past Spectrum-STI estimates^35,36,40^, and in HIV incidence estimates^41–44^, which reflects that both the estimated prevalence of syphilis in FSW and Yunnan’s key population size estimates are lower than in other countries^7,9,45,46^. The size estimate of FSW was based on self-identified sex workers; and almost certainly is an under-estimate of the total number of women who engage in transactional sex work. As a result, the relative share of FSW and MSM in incident cases is likely greater than 9.3% − and for MSM closer to their share in national case notifications in China and other countries^47,48^. In addition, FSW and MSM, by having on average relatively many partners, contribute more newly transmitted infections per episode, than lower-risk individuals.

The strengths of this study lie in the large number of data points identified from different populations and sources in Yunnan, the engagement of the Yunnan CDC in interpreting data and assigning weights to the prevalence studies used, and the use of Spectrum-STI to generate trend estimates. Spectrum-STI provided a unified coherent approach for synthesizing data from different sources, taking into account varying diagnostic tests, data collection methods and possible biases in sampling.

The provincial results, however, are very dependent on the underlying prevalence data, which vary in quantity, quality and representativeness. The sensitivity analysis showed how the trends were influenced by the data sources included, and the benefits from combining and triangulating multiple data types. The trend estimations for Yunnan show the benefits of extracting and reviewing data from different population groups and collected for different purposes. For lower-risk women the data from ANC sentinel surveillance and blood donor screening are similar. The routine ANC data suggested an increase, but this trend may have been biased by the change in testing regimen and expansion in coverage of screening service. For FSW and MSM there were marked differences between the estimates based on the provincial sentinel surveillance data and those based on the small-scale ad hoc studies. The former was, on average, much lower, which may reflect the fact that individuals who are part of the sentinel surveillance system are more likely to be screened and treated and as a result have, on average, a shorter duration of infection.

Our analysis focused on four population groups - however, this is a simplification of the real world and ignores other key populations (e.g., Intravenous drug users) and populations at intermediate risk of infection (e.g., truck drivers, migrants and mining workers and male clients of FSW^49–52^), and even with the identified risk groups of FSW and MSM, there are sub-groups with different risk profile and prevalence. As additional data become available (both prevalence data and population size) the model for Yunnan should be expanded. Incorporating other groups – e.g., an intermediate-risk groups, who were likely under-sampled in the studies we have included (e.g. of ANC women), would likely increase the prevalence estimates compared to those currently presented.

Yunnan has a strong sentinel surveillance system focused on FSW and MSM and on syphilis in ANC women. Future estimates would benefit from an expansion of these efforts to collate additional data from lower-risk men and populations at intermediate risk of infection, and from assessing how representative the sentinel surveillance data are of the overall FSW and MSM populations. In addition, estimates would benefit from additional studies looking at the relative size of the different population groups modelled.

Tools like Spectrum-STI have a role to play in helping program managers interpret surveillance data and monitor progress towards reducing the public health burden of STIs and the elimination of mother-to-child transmission of syphilis. Spectrum-STI provided a constructive framework for collating and analyzing data in Yunnan from a variety of different data sources and encouraged dialogue between the key players. The resulting estimates suggests that the prevalence of syphilis in the general population in Yunnan is low, under 0.30% in men and women 15 to 49 years. Syphilis, nevertheless, continues to pose a public health challenge, and prevention, screening and surveillance efforts would benefit from additional strengthening and expansion beyond the current target groups.

## Data Availability

All data analyzed and used, and all estimates generated during this study are included in this published article and its Tables.
